# NMR Characterization of the Near Native and Unfolded States of the PTB Domain of Dok1: Alternate Conformations and Residual Clusters

**DOI:** 10.1371/journal.pone.0090557

**Published:** 2014-02-28

**Authors:** Sebanti Gupta, Surajit Bhattacharjya

**Affiliations:** School of Biological Sciences, Division of Structural and Computational Biology, Nanyang Technological University, Singapore, Singapore; MRC National Institute for Medical Research, United Kingdom

## Abstract

**Background:**

Phosphotyrosine binding (PTB) domains are critically involved in cellular signaling and diseases. PTB domains are categorized into three distinct structural classes namely IRS-like, Shc-like and Dab-like. All PTB domains consist of a core pleckstrin homology (PH) domain with additional structural elements in Shc and Dab groups. The core PH fold of the PTB domain contains a seven stranded β-sheet and a long C-terminal helix.

**Principal Findings:**

In this work, the PTB domain of Dok1 protein has been characterized, by use of NMR spectroscopy, in solutions containing sub-denaturing and denaturing concentrations of urea. We find that the Dok1 PTB domain displays, at sub-denaturing concentrations of urea, alternate conformational states for residues located in the C-terminal helix and in the β5 strand of the β-sheet region. The β5 strand of PTB domain has been found to be experiencing significant chemical shift perturbations in the presence of urea. Notably, many of these residues in the helix and in the β5 strand are also involved in ligand binding. Structural and dynamical analyses at 7 M urea showed that the PTB domain is unfolded with islands of motionally restricted regions in the polypeptide chain. Further, the C-terminal helix appears to be persisted in the unfolded state of the PTB domain. By contrast, residues encompassing β-sheets, loops, and the short N-terminal helix lack any preferred secondary structures. Moreover, these residues demonstrated an intimate contact with the denaturant.

**Significance:**

This study implicates existence of alternate conformational states around the ligand binding pocket of the PTB domain either in the native or in the near native conditions. Further, the current study demonstrates that the C-terminal helical region of PTB domain may be considered as a potential site for the initiation of folding.

## Introduction

The funnel shaped energy landscape of folded proteins displays distribution of conformational states whereby the natively folded state of a protein mostly occupies the lowest energy level. Conversely, other conformational states of protein including partially folded and unfolded states reside at the high energy region of the folding funnel landscape. The folded conformation of a protein, largely populated under physiological solution condition, often undergoes excursion to other low-lying energy states that surrounds the free energy minimum[1], [2], [3], [4]. These alternate conformations, largely folded in nature, have been implicated in ligand binding, substrate recognition and response to the changes of environmental conditions[1], [3], [4], [5], [6]. Whereas, partially folded and unfolded states of a protein are of significance for a number of reasons. The unfolded state of a protein has been considered as a starting point for its refolding under *in vitro* condition whereas proteins those are natively unfolded contain important biological functions[7], [8], [9], [10], [11], [12]. Therefore, characterization of alternate structural states of proteins at atomic resolution has been thought to be central for the appropriate description of the energy landscape [7], [13], [14], [15], [16]. NMR spectroscopy serves as an essential tool for the structural and dynamical studies of alternate conformations of proteins[17], [18], [19], [20]. The non-linear temperature dependence of amide proton chemical shift has been attributed to the lowly populated alternate conformational states of folded proteins [21], [22]. Such alternate conformational states are separated by an energy barrier of 2–3 kcal/mol from the native state and can be modulated under mildly denaturing conditions[5], [22], [23]. Unfolded and partially folded states of single domain proteins have been characterized by use of NMR methods[24], [25], [26], [27], [28], [29], [30], [31], [32]. These studies have found short-range and also, in few cases, long-range structural order in the highly unfolded states of proteins[26], [32], [33], [34], [35]. Common type of ordering includes existence of nascent secondary structures[32], [35], [36], [37], localized or short range hydrophobic clusters[26], [38], [39], [40]. NMR techniques, paramagnetic relaxation and residual dipolar coupling, indicated probable proximity among amino acids located far in primary structures in these unfolded states[34], [41], [42]. The extent of residual structures in the unfolded states of proteins may be determined by the amino acid sequences and denaturing solution conditions. Replacements of critical hydrophobic core residues with multiple Ala have demonstrated stabilization of unfolded or partially folded states of proteins in the absence of chaotrope[36], [41], [43]. Several structural studies showed that these unfolded states are somewhat more structured and compact as compared to the unfolded or partially folded states obtained in denaturing solutions[7], [36].

Folding and unfolding studies of protein interaction domains have generated valuable insights toward the protein folding mechanisms [44], [45]. Notable examples include extensive high-resolution folding analyses of a number of SH3 domains by various research groups[24], [46], [47], [48], [49]. From the functional perspective, cell signaling by SH3 domains can be mediated by the recognition of proline rich sequences in their binding partners. Whereas SH2 and PTB domains, despite of different global fold, bind to phosphotyrosine containing motifs in target proteins for signal transduction processes[50], [51], [52], [53]. In general, folding/unfolding studies of phosphotyrosine binding domains like SH2, PTB are relatively scarce[54], [55]. PTB domains are categorized into three structural families: IRS-like, Shc-like and Dab-like [50]. The IRS-like and Shc-like PTB domains recognize a conserved sequence motif NPXY of phosphorylated tyrosine, whereas Dab-like PTB domains bind to the non-phosphorylated protein ligands. The global fold of all three types of PTB domains shares a core structural PH-like domain consisted of seven antiparallel β-strands packing against a long C-terminal helix. There is also either a short or long N-terminal helix in IRS-like PTB and Shc/Dab PTB, respectively. The mammalian Dok protein family (Dok1 to Dok7) functions as an adaptor molecule characterized by the presence of a conserved PTB domain along with a PH domain [51]. The PTB domain (residues 154–252) of Dok1 mediates protein-protein interactions by binding to the phosphotyrosine containing motifs of proteins including RET and β cytosolic tails of integrins[56], [57], [58]. Crystal structures of the Dok1 PTB domain have revealed IRS-like fold with a ligand binding pocket maintained along the β5 strand of the seven stranded β-sheet and the C-terminal helix[57]. In this work, we have investigated, by use of NMR spectroscopy, urea induced unfolded states of the PTB domain of human Dok1 protein. We have also examined conformational fluctuations of the native structure either in presence of β3 tail or at sub-denaturing concentrations of urea.

## Results

### Urea Induced Unfolding of Dok1 PTB Domain

**Figure 1:**
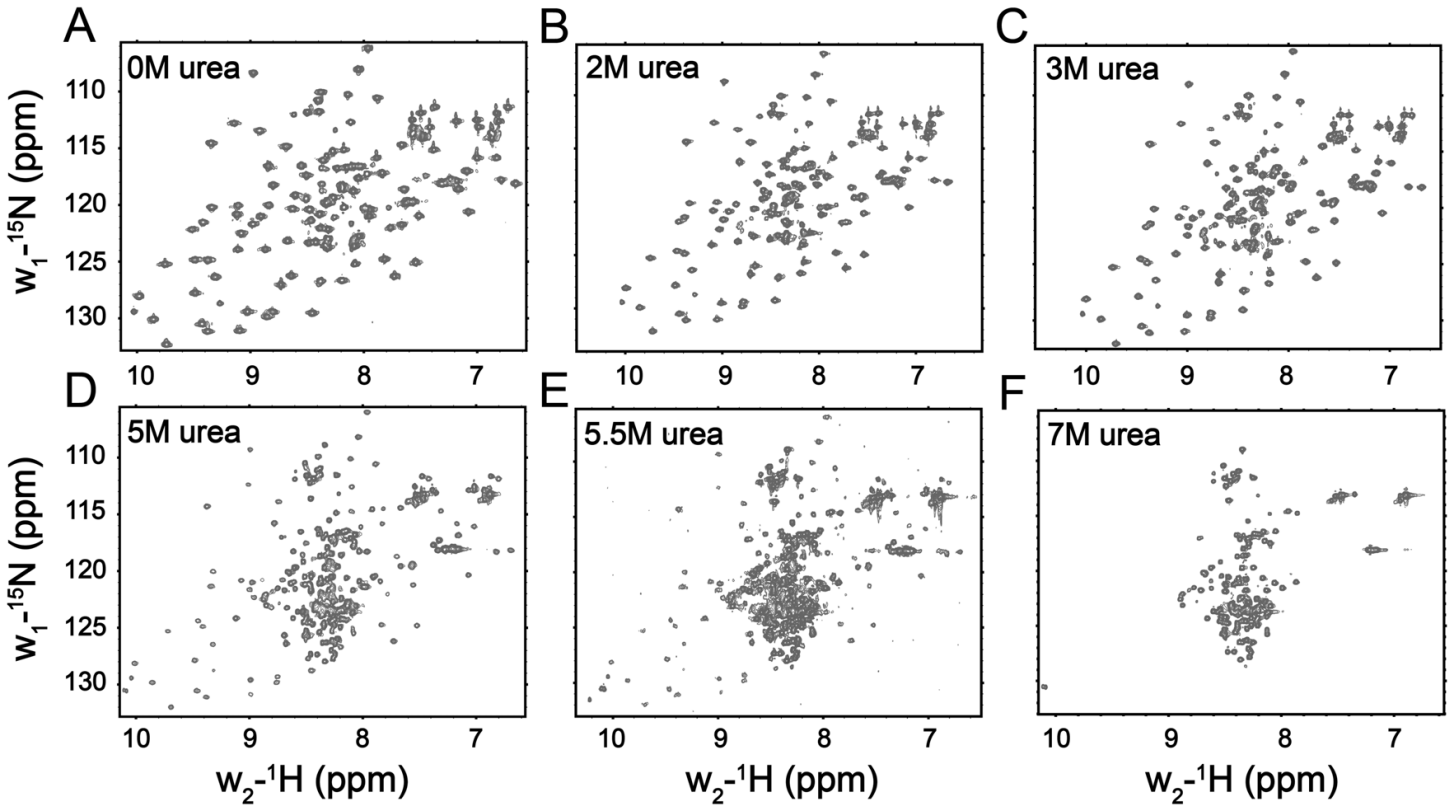
Urea-induced Unfolding of the Dok1 PTB Domain: ^15^N-^1^H HSQC spectra of the Dok1 PTB domain at different concentrations, 0 M (panel A), 2 M (panel B), 3 M (panel C), 5 M (panel D), 5.5 M (panel E) and 7 M (panel F), of urea.

**Figure 2:**
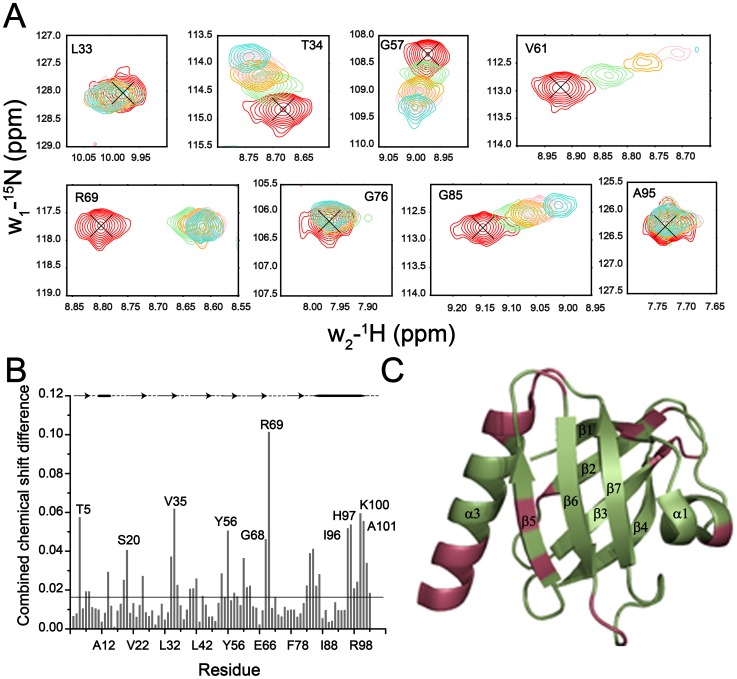
Localization of Urea Binding to the Dok1 PTB Domain: (panel A) Sections of ^15^N-^1^H HSQC spectra of representative residues of the PTB domain showing urea dependent chemical shift changes in 0 M (red contour), 2 M (green contour), 3 M (yellow contour), 4 M (pink contour), 5 M (cyan contour) urea. As can be seen, some of the residues e.g. T34, G57, V61, R69, G85 show pronounced chemical shift changes in HSQC spectra. (panel B) A bar diagram showing combined chemical shift difference of ^15^N and HN resonances of each residue of the PTB domain between native and 1 M urea. A line drawn at 0.018 ppm indicates average changes of the chemical shift. The secondary structural elements are shown at the top (panel C) Ribbon representation of the three dimensional structure of the Dok1 PTB domain (pdb: 2v76) highlighting residues (in red) those showed an above average combined chemical shift change in 1 M urea. The figure is prepared using PyMOL program.

The ^15^N-^1^H HSQC spectrum of the PTB domain is well dispersed, as expected for a folded protein (Fig. 1A). HSQC cross-peaks were assigned using standard triple resonance experiments and further confirmed with the aid of previously reported chemical shifts of the PTB domain of Dok1 (BMRB accession number 15551) [57]. ^15^N-^1^H HSQC spectra of the PTB domain were acquired at various concentrations, ranging from 0.25 M to 7 M, of urea. The native population of PTB domain predominated upto 2 M urea concentration as judged by the wide chemical shift dispersion and paucity of new cross-peaks corresponding to the unfolded state (Fig. 1B). At 3 M concentration of urea, a few new ^1^H-^15^N correlations were observed at the center of the HSQC spectrum (Fig. 1C), possibly indicating initiation of the denaturation. Inspection of the ^15^N-^1^H HSQC spectra of the PTB domain at 5 and 5.5 M urea have revealed a significant reduction in intensity of the native state HSQC resonances with concomitant appearance of signals from unfolded species (Fig. 1D and Fig. 1E). At 7 M urea, there were no detectable HSQC cross-peaks corresponding to the native conformation of the PTB domain (Fig. 1F). The HSQC spectrum at 7 M urea delineates a narrow dispersion of the amide proton (1 ppm) and ^15^N chemical shifts (Fig. 1F), characteristics of the unfolded state of a protein [26], [32], [59]. We have observed that HSQC cross-peaks of some residues showed a progressive chemical shift changes upon additions of urea (Fig. 2A). The urea-PTB domain interactions were found to be on the fast exchange in terms of chemical shift time scale, indicating a low affinity interaction between the protein and denaturant. Figure 2 (panel B) shows a bar diagram of combined chemical shift changes of ^15^N and ^1^HN in 1 M urea. Notably, residues T5, R14, S20, V25, T34, V35,L42, R54, Y56, V61, G68, R69, A83, Q84, G85, N86, D87, I96, H97, R98, Q99, K100, A101, Q102 have experienced a relatively higher chemical shift changes (≥0.02 ppm), in comparison to the other (Fig. 2B). The magnitude of chemical shift perturbation was found to be higher (≥0.03 ppm) at 3 M urea for these residues (Fig. S1). A solvent accessible surface calculation of the PTB domain structure showed that these residues are solvent exposed, and therefore, may be accessible for interactions with urea. Such interactions could be mediated by hydrogen bond formations between the polar groups of urea and with the backbone or sidechains of the polar amino acids. The urea-induced chemical shift perturbations are mapped onto the structure of PTB domain (Fig. 2C). As can be seen, a part of the C-terminal helix and the β5 strand of the β-sheet have contributed mostly to urea binding (Fig. 2C).

### Temperature Dependence of the Amide Proton Chemical Shift at Sub-denaturing Concentrations of Urea

**Figure 3:**
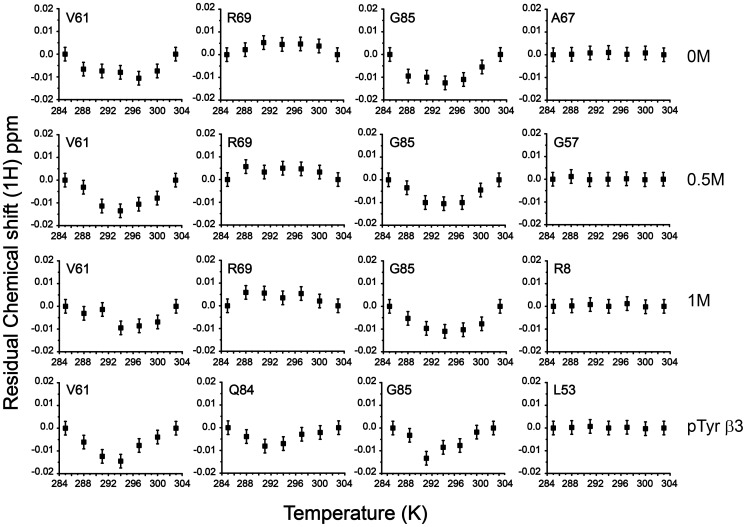
Non-linear Profiles of the Amide Chemical Shifts with Temperature of the Dok1 PTB domain in Native, in Presence of Phosphorylated β3 Cytosolic Tail and in Sub-denaturing Conditions: Representative examples of residues of the Dok1 PTB domain showing non-linear or curved temperature profile in temperature dependent changes of the amide proton chemical shifts in 0 M, 0.5 M, 1 M urea and with β3 phosphorylated cytosolic tail of integrin. The right panel shows a few examples of residues delineating linear temperature dependence. Error bars indicate chemical shift measurement error, which was estimated to be ±0.003 ppm.

**Figure 4:**
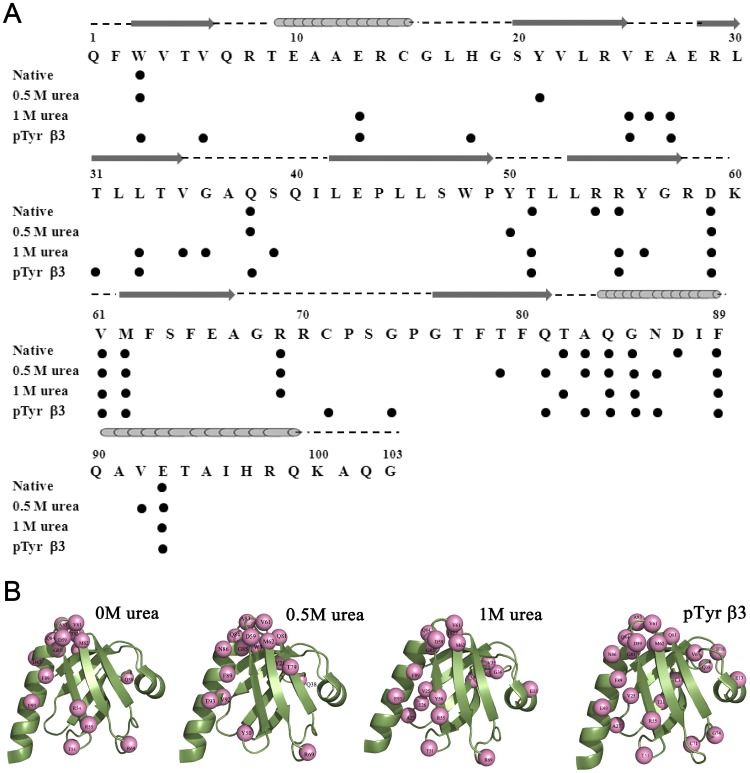
Residues of the Dok1 PTB Domain with Curved Temperature Profiles: (panel A) A Summary of the residues showing curved temperature dependence of the Dok1 PTB domain in native, in urea: 0.5 M, 1 M concentration and native PTB in complex with phosphorylated β3 cytosolic tail. The filled circle represents residues displaying non-linear behavior. The secondary structures of the native Dok1 PTB domain are shown above amino acid sequence. (panel B) Ribbon representations of the three-dimensional structure of the Dok1 PTB domain (pdb: 2v76) highlighting (in sphere) residues showing curved temperature profiles in native, 0.5 M, 1 M urea concentrations and native in complex with phosphorylated β3 cytosolic tail. Residues showing non-linear behavior in temperature dependent amide proton chemical shift changes are excursing alternate conformational states from their native conformations.

The chemical shift of amide proton usually demarcates a linear dependence as a function of temperature[60]. However, a non-linear change in chemical shift of amide proton has been attributed to plausible sampling of alternate conformational states of high energy[5]. We have examined temperature depended of the amide proton chemical shift of residues of the Dok1 PTB domain, at native and near native conditions, to gain insight into alternate conformations. The Dok1 PTB domain binds to phosphorylated peptide ligands including the cytosolic tail of different β-integrins[57]. We have further investigated alternative conformational states of the PTB domain in complex with phosphorylated peptide ligand. Toward this, a series of ^15^N-^1^H HSQC spectra of the PTB domain were obtained at seven different temperatures (285 K, 288 K, 291 K, 294 K, 297 K, 300 K and 303 K) at four different conditions including 0 M urea or native, in the presence of phosphorylated β3 tail and in sub-denaturing concentrations, at 0.5 M and 1 M, of urea (Fig. S2). Note, at these urea concentrations no detectable unfolding was observed for the PTB domain (Fig. 2). Most of the amide protons of the PTB domain showed linear temperature dependence as expected, however, there were as many as 16 residues delineating non-linear or curved temperature dependence profile under native condition. With a few exceptions, residues showing curved temperature profiles in 0 M urea also retained similar behavior in the presence of urea and phosphorylated β3 tail. Figure 3 shows representative examples of the non-linear temperature dependence for some selected residues. For a better illustration, residuals are also shown for residues, R8, L53, G57 and A67, experiencing a linear temperature profile (Fig. 3, right panel). Notably, more residues appear to show curved temperature dependence both in urea containing solution and in the presence of the peptide ligand (Fig. 4A). We have also noticed interesting trends in temperature dependence at native and in the presence of urea or ligand. Residues R54, D87 showed curved temperature dependence only in the absence of urea or ligand (Fig. 4A). Curved temperature profiles can be seen for some residues only at a single concentration of urea or in the presence of β3 tail. Residues e.g. W3, Q38, T51, R55, V61, M62, A83, Q84, G85 exhibited curved temperature profile in native and in either 0.5 M or 1 M urea solutions. In addition, certain residues from the distal β sheet, e.g. E13, V25, A27 and L33 have retained non-linear profile in the presence of phosphorylated β3 tail and in 1 M urea. Residues delineating curved temperature dependence are highlighted onto the structure of the Dok1 PTB domain (Fig. 4B).

### Conformational Characterization of the Dok1 PTB Domain in 7 M Urea

**Figure 5:**
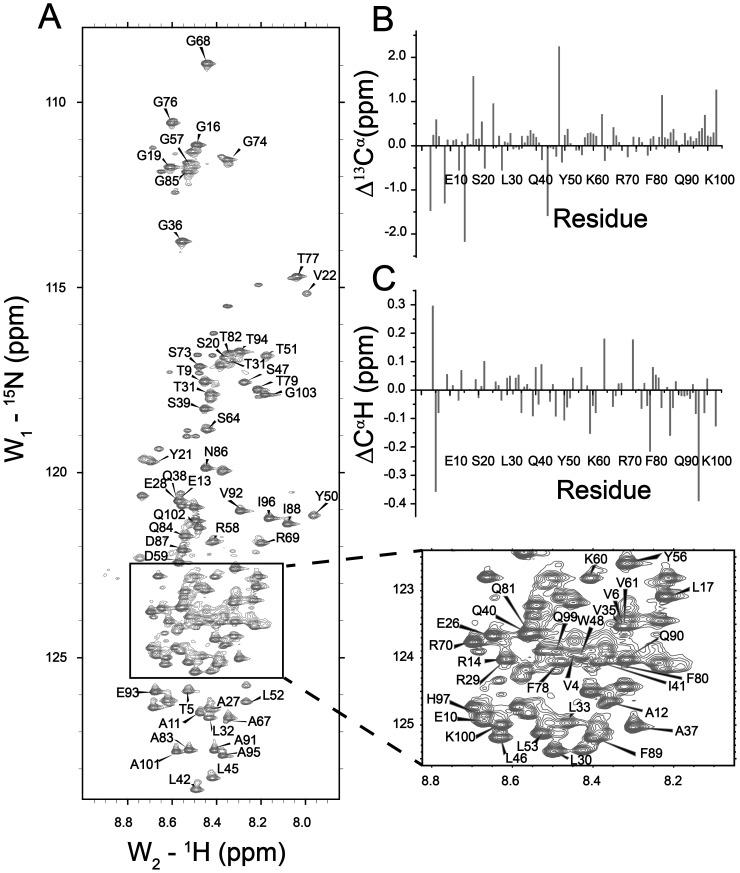
Conformational Propensities of the Unfolded State of Dok1 PTB Domain: (panel A) ^15^N-^1^H HSQC spectra of the Dok1 PTB domain in 7 M urea showing assignment of HSQC cross-peaks obtained through triple resonance experiments. A region of the ^15^N-^1^H HSQC spectrum has been shown in a box due to resonance crowding. ^15^N-^1^H HSQC cross-peaks which cannot be assigned, due to the overlapping resonances or for the lack of sequential connectivity in 3D spectra, are not marked in the spectrum. (panels B and C) Bar diagrams showing secondary chemical shift (deviation from random coil values) of ^13^C^α^ (panel B) and ^α^H (panel C) of each residue of the Dok1 PTB domain in 7 M urea.

The ^1^H-^15^N HSQC spectrum of Dok1 PTB domain in 7 M urea is shown in Figure 5A. The narrow resonance dispersion of the amide proton chemical shift of the spectrum within 1 ppm indicates unfolded conformation of the protein. However, the chemical shift dispersion of the ^15^N was sufficiently large for the assignment of the HSQC correlations under denaturing condition[28]. Backbone resonance assignments were achieved by a combined analyses of HNCA, HN(CO)CA, HNCACB and CBCA(CO)NH spectra. Sidechain resonances were identified using 3D ^1^H-^15^N NOESY-HSQC and TOCSY-HSQC spectral analyses. ^15^N-^1^H HSQC cross-peaks could be unambiguously identified for 86 residues. HSQC cross peaks cannot be assigned for some residues including F2, W3, R8, L23, R24, V25, E43, M62, F63, F65 and E66, possibly due to overlapping resonances and/or conformational exchange broadening. Regardless, resonance assignments (≥84%), in 7 M urea, encompass all of the major secondary structural elements allowing us to probe the structural and dynamical characterization of the unfolded state of the PTB domain.

Secondary structural propensity of the unfolded states of Dok1 PTB domain has been assessed from secondary chemical shifts and nuclear Overhauser effect (NOE) data. Secondary chemical shifts of ^13^C^α^ (Fig. 5B) and ^α^H (Fig.5C) were estimated after sequence dependent corrections[61], [62]. The secondary shifts for ^13^C^α^ and ^α^H were found to be rather limited due to the predominant population of the unfolded conformations in 7 M urea. The secondary shift for ^13^C^α^ and ^α^H delineated deviations of <0.5 ppm and <0.1 ppm, respectively, for most of residues, with few exceptions (Fig. 5B and Fig. 5C). Similar observations have been made in conformational analyses of unfolded proteins [28], [39], [63], [64]. The presence of positive (downfield) shift of ^13^C^α^ and the negative (upfield) shift of ^α^H indicates population of alpha helical conformations while the opposite trend has been observed for the β strand structural propensity [65], [66]. As can be seen, a number of continuous regions, including V35-I41, Y50-L52, G57-V61, A83-F89 and A91-G103, of Dok1 PTB domain demonstrated positive deviations of ^13^C^α^ chemical shift (Fig. 5B). Residues L42-S47 and R54-Y56 demarcated negative deviations of ^13^C^α^ chemical shift in 7 M urea (Fig. 5B). By contrast, stretches of residues showing similar ^α^H deviations were found to be limited (Fig. 5C). As such, stretches of residues Y50-L52, D59-V61, D87-T94, I96-Q99 have been detected with negative deviations in ^α^H shifts (Fig. 5C). As noted in earlier studies, the ^α^H chemical shift of unfolded proteins generally lies very close to the random coil values due to lower deviation of ^α^H chemical shift in the secondary structures [19], [20]. Regardless, both the secondary chemical shifts, ^13^C^α^ and ^α^H, indicated that residues Y50-L52 and residues D59-V61Might have helical propensity (Fig. 5B and Fig. 5C). These residues are located at the loops, adjacent to the β5 strand, of the native structure of the PTB domain. More strikingly, at the C-terminus region of the PTB domain, there are continuous stretches of residues, A83-F89 and A91-G103, with positive deviations of ^13^C^α^ chemical shift (Fig. 5B). Along with that concomitant negative deviations of ^α^H chemical shift for the residues Q84, D87, I88, Q90-T94, I96-H97, Q99, A101 and G103, can be detected (Fig. 5C). These observations indicate populated helical conformations at the C-terminus of the PTB domain in 7 M urea. These residues encompass the long C-terminal helix (Q90-Q102) and the adjacent loop (Q84-F89) of the native Dok1 PTB domain. Analyses of backbone conformational propensities using ^13^C^β^ chemical shift deviation were found to be rather ambiguous particularly for the stretch of helical conformations (data not shown). In a previous study, the secondary shift of ^13^C^β^ has also been seen to be a poor indictor of the secondary structure for the denatured protein [35].

**Figure 6:**
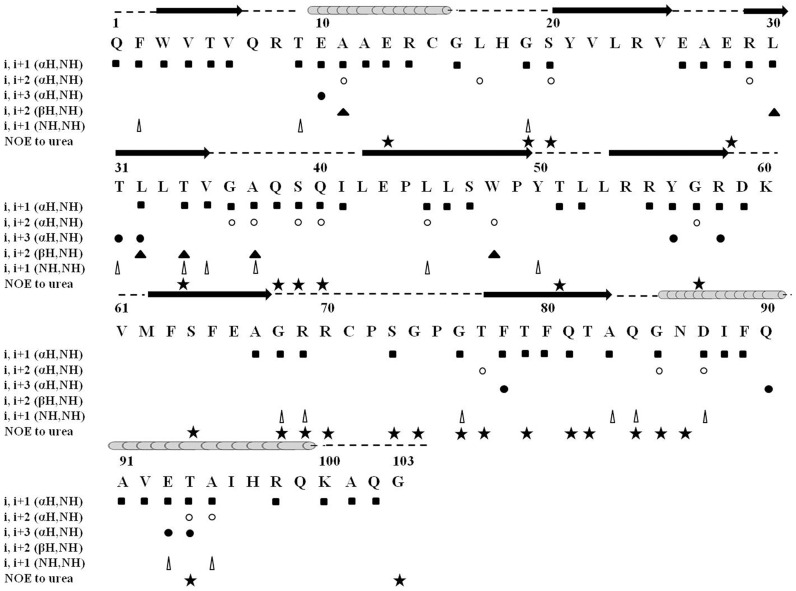
Analyses of the nuclear Overhauser effect (NOE) Spectra of the Unfolded State of Dok1 PTB Domain: Summary of NOE contact identified in ^15^N-edited 3D-NOESY-HSQC spectra of the PTB domain in 7 M urea. NOEs between the amide protons of residues of PTB domain with urea protons have also been indicated. The secondary structures of the native Dok1 PTB domain are shown above amino acid sequence.

Conformational propensities of the denatured states of the PTB domain were further evaluated by nuclear Overhauser effect (NOE) spectra. Analysis of 3D ^15^N NOESY-HSQC revealed intense sequential NOEs of ^α^H/NH throughout the sequence. Although, dispersion of the ^1^H chemical shift is limited in 7 M urea, some sequential NH/NH NOEs could be detected along with a few medium range NOEs (Fig. 6). All together 16 ^α^H/NH (i, i+2), 9 ^α^H/NH (i, i+3) and 6 ^β^H/NH (i to i+2) NOEs were identified for the Dok1 PTB domain in 7 M urea. The C-terminal region of the PTB domain contains several of these medium range and NH/NH NOEs, indicating preference for helical conformations. By contrast, other residues of the PTB domain in the denatured state appear to be sampling the extended region of the φ, ψ conformational space as suggested by the strong sequential ^α^H/NH NOE connectivities. Interestingly, we have observed as many as 26 NOE contacts involving urea and PTB domain (Fig. 6, Fig. S3). Majority of these residues displaying NOE contacts with urea are polar and charged. These data may indicate a preferential solvation of hydrophilic residues, over hydrophobic residues, by the denaturant.

### Dynamical Characterization of the Denatured State of Dok1 PTB in 7 M Urea

**Figure 7:**
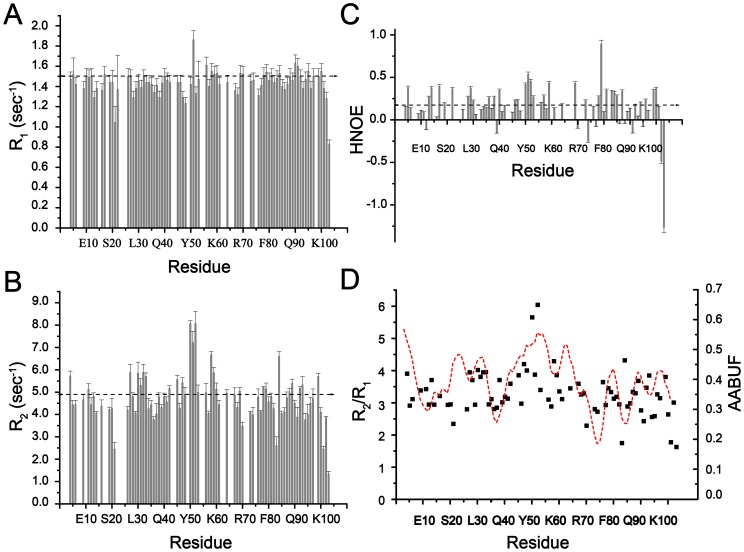
Dynamical Characteristic of the Unfolded State of Dok1 PTB Domain. Plots show measurements of ^15^N relaxation parameters R_1_ (panel A), R_2_ (panel B) and heteronuclear {^1^H}-^15^N NOE (panel C) of residues of the PTB domain of Dok1 in 7 M urea. (panel D) Bar diagram showing ratio of R_2_/R_1_ and AABUF vs residue of the PTB domain of Dok1. The peaks in the plot delineate residues those are highly buried in the non-polar core of the PTB domain. The mean value of the relaxation parameters, in the panel A, panel B and panel C, has been indicated by a dotted line.

Backbone dynamics of the unfolded states of the PTB domain were investigated by measuring relaxation parameters, R_1_ (1/T_1_), R_2_ (1/T_2_) and heteronuclear {^1^H}-^15^N NOE (Fig. 7). The heteronuclear NOE data presented in this work may be considered as qualitative due to the short value of the inter-scan delay used in this experiment[67]. We have also estimated relaxation parameters for the native PTB domain for comparison with the unfolded conformations (Fig. S4). The relaxation characteristics, in particular R_2_ and heteronuclear {^1^H}-^15^N NOE, of the native and unfolded states of the PTB domain delineated marked differences, indicating sensitivity of these parameters for global conformational changes (Fig. S4). Further analyses of the dynamics of the native PTB domain alone and the complex with peptide ligand will be presented elsewhere. As can be seen, R_1_ values of the denatured states of the PTB domain span from 0.83 to 1.86 sec^−1^ with an average of 1.44 sec^−1^ (Fig. 7A). Conversely, the heteronuclear {^1^H}-^15^N NOE values delineate a much wider variation ranging from −1.26 to +0.8 (Fig. 7C). The observed heteronuclear NOE values were well below the cutoff for the folded proteins (+0.81)[68], [69] implying flexibility throughout the urea unfolded states of the PTB domain of Dok1. The R_2_ values are also found to be exhibiting a wider variation;1.34 to 8.05 sec^−1^ with an average of 4.76 sec^−1^ (Fig. 7B). Further analyses of R_1_ and R_2_, in terms of R_2_/R_1_, discern non-random structures in the urea denatured states of the protein (Fig. 7D). As can be seen, there are three maxima or peaks in the R_2_/R_1_ plot representing clusters of residues involving residues A27-V35, L45-L53 and Y56-V61. Such clusters have been observed for urea unfolded states of other proteins [26], [35], [39] and also for the natively unfolded proteins[38], [70]. These observations have been interpreted as potential mutual interactions among the residues within the cluster or between the clusters[26], [35], [39].

**Figure 8:**
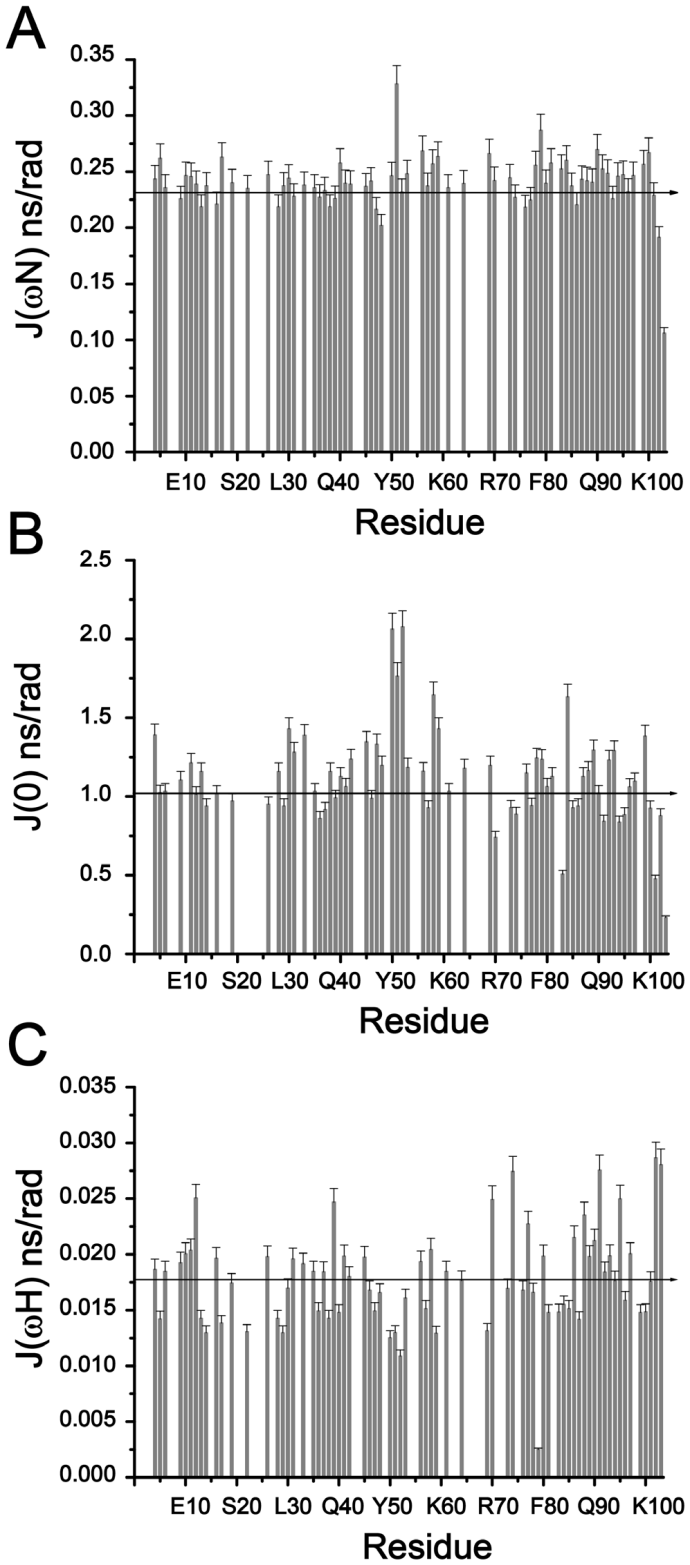
Spectral Density Characteristics of the Unfolded State of Dok1 PTB Domain. Plots showing determination of reduced spectral density functions J(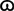
_N_) (panel A), J(0) (panel B) and J(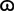
_H_) (panel C) of the Dok1 PTB domain in 7 M urea. The mean value of the spectral density functions has been indicated by a solid line.

Relaxation data were additionally analyzed using reduced spectral density mapping. The spectral density functions, J(0), J(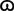
_N_) and J(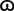
_H_), were determined (see Experimental Procedure) and plotted in Figure 8. The J(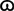
_N_) parameter, largely determined by R_1_, shows similar values across the polypeptide chain, except for the last two residues, Q102 and G103, at the C-terminus (Fig. 8A). J(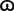
_N_) for these residues Q102 and G103 are diminished (Fig. 8A). The values for J(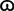
_H_), mostly determined by heteronuclear NOE, represent a wider variation across the polypeptide chain whereby higher values are observed for residues, Q102 and G103, at C-terminus along with fewer residues including A12, S39, R70, G74, A91, A95 at the middle and the N-terminus (Fig. 8C). The higher J(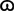
_H_) of residues A12, S39, R70, G74, A91, A95 indicate a fast motion without any associated contribution to intermediate time scale motions (Fig. 8C). The J(0) spectral density function, mainly dictated by R_2_, shows a much larger variation, with a maximum and minimum of 2.07 and 0.23 ns/rad, respectively, across the amino acid sequence. The C-terminal residues A101-G103 are characterized by below average (<1 rad/sec) J(0) values. There are also a few residues, R70, S73, G74, T77, A83, G85, A91, T94, A95 at the C-terminus with below average J(0) value (Fig. 8B). The sequence variation of J(0) between residues E28-K60 reveals that there are three regions with above average J(0) ranging from 1.3 to 2.0 ns/rad (Fig. 8B).

## Discussion

### Structural and Dynamical Changes of the Dok1 PTB Domain at Sub-denaturing Concentrations of Urea

**Figure 9:**
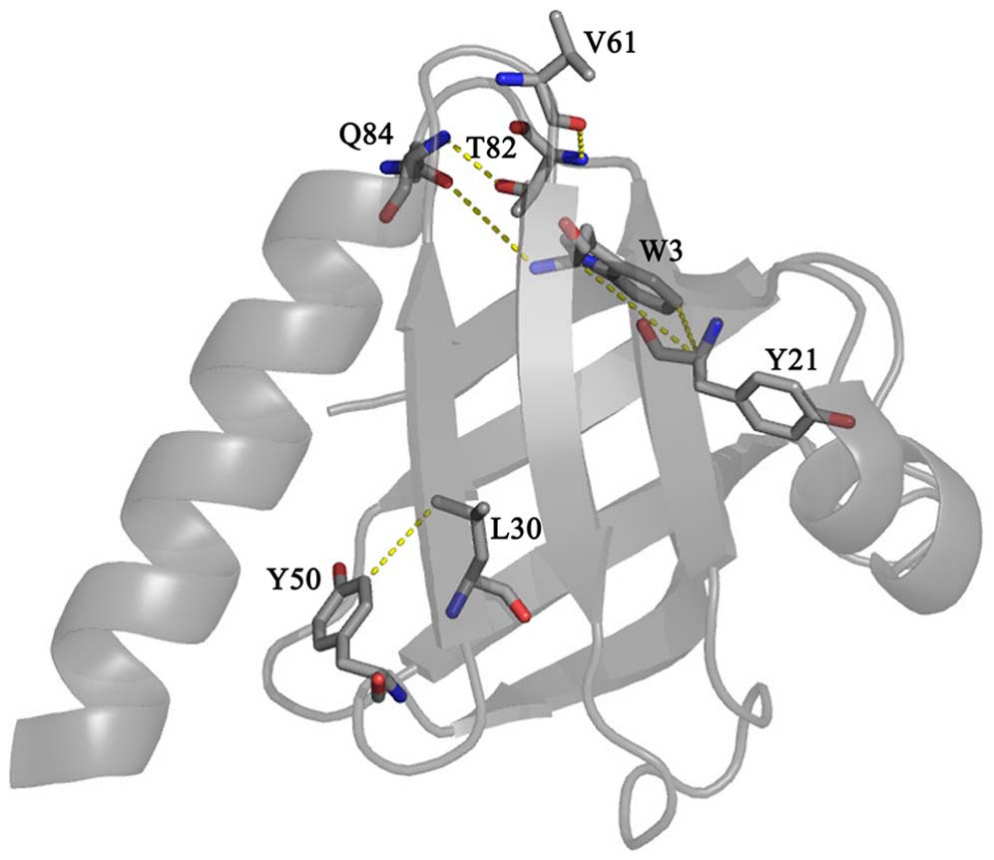
Structural Origin of Non-linear Temperature Dependence: Ribbon representation of the three-dimensional structure of the PTB domain showing close contact among residues involved in non-linear temperature dependence of the amide proton chemical shifts.

Protein domains essentially act as a molecular interface for their interactions with nucleic acids, proteins and phospholipids. These binding events in turn control different cellular processes from signal transduction to transcription regulation[71]. It is already well recognized that the function of a protein may be closely linked to its structure through the fine tuning of internal dynamics. In response to a signal (input), the intrinsic dynamical property of a protein could be altered with the concomitant shift among different fluctuating energy states[1]. The complexity of such interactions is often determined by the flexibility of the binding site and structural rearrangements that would occur upon ligand binding. In order to gain insight about the protein-ligand interactions, energy landscape approach introduces a conceptual framework of combinatorial consideration based on the advances in theoretical and experimental observations[72], [73], [74]. Under the near native folding conditions and during the signaling events, the energy landscape may remain populated with numerous structurally similar and energetically closer alternative conformations [23], [73], [75]. Consequently, the near native conformations and interactions mechanism have been considered to be quite overlapping in nature[73], [76]. These alternative conformational states are the major determinants of the ruggedness of the energy landscape that might play critical roles in folding, binding and stability of protein molecules [3], [6], [23], [77]. These excited states of proteins are also important for the adaptability at different environmental conditions including temperature, pH, chemical denaturants and pressure[5], [23]. The existence of non-linear temperature dependence of amide proton chemical shift is an indication of accessible alternative conformational states[21], [22]. These locally unfolded alternative conformational states have been shown to be composed of approximately five residues centering on the residue exhibiting curved temperature dependence[21], [22], [78]. In the presence of sub-denaturing concentrations of urea, other alternative states can be seen as the denaturant may decrease the free energy difference between the native and the excited states[5], [22]. The native Dok1 PTB domain shows as many as 16 residues with non-linear pattern of temperature dependence (Fig. 3). Most of these residues, except residue W3 and Q38, are proximal or located at the ligand binding site of the PTB domain. Most notably, residues R55, Y56 and R69, exhibiting curved temperature profile, demarcate direct interactions with the phosphate group of the peptide ligands [57]. In addition, non-polar residues of the PTB domain involved in binding with the phosphorylated ligand also show non-linear temperature dependence of amide protons (Fig. 4B). With increasing concentrations of urea, many more residues of the PTB domain with alternative conformations were observed. At 1 M urea, residues E13, V25, E26, A27, L33, V35, G36 and S39 have responded in a non-linear fashion with temperature (Fig. 4B). Interestingly, in presence of phosphorylated β3 tail non-linear responses were also observed for residues, V6, E13, H18, V25, A27, T31, L33 and Q38, located far from the binding pocket (Fig. 4B). However, it cannot be ascertained, based on curved temperature profile, about the number of alternate conformational states involved in the process. There could be more than two conformational states undergoing simultaneous structural fluctuations. Temperature dependence of the amide proton chemical shift identifies regions in protein experiencing structural fluctuations. Regardless, we have looked for plausible structural origin of the non-linear temperature dependence. Interestingly, the side chains of residues Q84 and W3 are in close contact in the 3D structure of the PTB domain. Also, in this region side chains of residues T82 and V61 are engaged in complementary packing interactions (Fig. 9). Similarly, aromatic rings of residues W3, Y21 and Y50 and alkyl side chain of residue L30 are proximally orientated (Fig. 9). It is likely that aromatic ring flipping and/or inter-sidechain hydrogen bonds could be responsible for local structural changes leading to curved temperature profiles. Further, we find interesting correlations between urea induced chemical shift perturbations and temperature dependence under near native conditions. As can be seen, an above average chemical shift perturbation has been estimated for residues involved in ligand binding including the C-terminal helix and the β5 strand of the PTB domain (Fig. 2). Taken together, residues engaged in ligand interactions appear to be accessing alternative conformations in the current study. It is rather tempting to speculate that these high energy conformational states might have implications in binding interactions with the phosphorylated peptides and proteins. Such conjecture has been further validated as residues in the binding pocket of the PTB domain access high energy states in complex with the phosphorylated β3 peptide. Notably, alternate conformational states for residues in the substrate binding sites are previously observed for other proteins including SUMO-1, ubiquitin and NEDD8[5], [79]. In these proteins, high energy conformational states are known to be critical for the recognition of substrates. Interestingly, in the presence of the ligand alternate conformations of a cluster of residues located in the distal β sheet of the PTB domain of Dok1 are unique in nature (Fig. 4B). Although, the mechanism by which the effect of peptide binding is transmitted to the remote cluster is not clear at present. Regardless, this finding may suggest plausible involvement of any allosteric regulation between these two sites. Notably, similar cluster of residues with non-linear temperature dependence has been observed in the presence of 1 M urea. Such resemblance in dynamical behavior of PTB domain at the near native state (1 M urea) and in the presence of its binding partner is striking and may indicate an overlapping nature of the near-native folding and binding mechanism.

### Unfolded States of the PTB Domain in 7 M Urea

^15^N-^1^H HSQC spectra of PTB domain acquired at various concentrations of denaturant revealed detectable binding of the denaturant with the folded protein. Despite a direct binding of urea, the folded conformation of the PTB domain appears to be stable and unfolds only at 7 M urea. The HSQC spectral changes, with low to high concentrations of urea, have indicated an unfolding transition whereby the folded and unfolded states are in slow exchange at the NMR time scale. The PTB domain of Dok1 assumes highly unfolded conformations at 7 M urea without any evidence of long-range interactions. A large number of residues including polar, charged and Gly demonstrated NOE contacts with the denaturant (Fig. 6, Fig. S3). It may be noteworthy that these urea-protein NOEs are predominantly localized among residues in the sequence S63-N86 that encompasses the β6-loop-β7 and beginning of the C-terminal helix (Fig. 6). This may further imply toward a preferred urea binding region of the unfolded states of the PTB domain. Additionally, it is probable that this region of the PTB domain may undergo a slow refolding event during dilution of the denaturant. Further, inter-molecular NOEs between urea and polar residues of the PTB domain corroborate well with a MD simulation study of a protein CI2 [80]. In this study, denaturant molecules show a longer residence time with the hydrophilic residues in comparison to the nonpolar residues [80]. In direct binding mechanism of denaturation, it has been postulated that the carbonyl oxygen atom of urea may form hydrogen bonds with the backbone amide protons of proteins. Further, side chains of the polar residues may also form additional hydrogen bonds with urea molecules stabilizing urea-protein interactions around hydrophilic residues. Collectively, such interactions might disrupt conformational integrity of the folded protein causing their unfolding. A recent study based on NMR, x-ray scattering and statistical coil modeling demonstrated that the direct binding of urea with the protein backbone is primarily responsible for urea induced denaturation [81]. Although the mode of action of urea as a denaturant is largely debated, the recent NMR studies including the present one and concurrent MD simulations data potentially indicate that a direct mode of urea binding to proteins may overwhelm its indirect action on water structure.

### Conformational and Dynamical Characteristic of the Unfolded States

As evident from a number of studies long-range interactions are largely absent in the unfolded states of proteins at a high concentration of denaturant[26], [32], [82], [83], [84]. Short-range or local interactions and intrinsic sequence propensity of the polypeptide chain appear to be the major determinant for the deviations from random-coil behavior in unfolded proteins. NMR parameters, secondary chemical shifts and NOEs, have revealed conformational characteristics of the PTB domain in 7 M urea. Intense sequential (^α^H/NH) NOEs and secondary chemical shift parameters indicated that the unfolded PTB domain is in largely extended conformations. The unfolded conformational ensembles of the PTB domain are also dynamically flexible as indicated by low value of hetero-nuclear NOEs. However, non-random conformations of the PTB domain can be seen at the C-terminal region and for a few short segments of the protein. The C-terminus of the PTB domain is sustained by a long helix encompassing residues N86-K100 in its native state. Residues in this segment of the PTB domain demonstrated a consensus pattern of helical conformations in secondary chemical shifts in 7 M urea (Fig. 5B and Fig. 5C). There were also medium range NOEs diagnostic of helical conformations (Fig. 6). However, we did not observe a continuous NOE connectivity of medium range NOEs, the secondary chemical shifts were also discontinuous along the segment e.g. residues G85, N86, A95. Thus, the population of such helical conformations could be low and may be nascent in nature. Relaxation studies indicated a complex dynamics for the residual structures at the C-terminal helical region. This has been evident by the presence of both positive and negative heteronuclear NOEs and above average R_2_ and below average low R_2_ values in the helical segment (Fig. 7). The below average J(0) and above average J(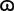
_H_) values in spectral density mapping were also indicative of fast motion whereas other residues, with higher J(0) and lower J(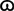
_H_), may be experiencing motional restriction at sub-nanosecond timescale. The β-sheet region of the Dok1 PTB domain in 7 M urea did not exhibit any preferred secondary conformations expect for residues at the loops connecting β4, β5 and β6 strands. Most of the residues corresponding to the β-sheet region of the native PTB domain were also found to be motionally flexible, although non-random conformations and motional restriction can be seen for three short regions namely residues A27-V32, L46-L53 and Y56-V61. Above average values of R_2_/R_1_ and positive values of heteronuclear NOE have indicated formation of clusters with non-random motions. Further, the above average J(0) and below average J(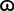
_H_) values in spectral density mapping correlate with a relatively slower motion of these clusters in 7 M urea. Such slow motions may also indicate plausible conformational exchange processes contributing to the higher R_2_ values. Notably, most of the residues in these clusters are non-polar or aromatic and did not exhibit NOEs to urea. This may perhaps explain non-random characteristics of these hydrophobic clusters. A plot of the average area buried upon folding (AABUF) of the domain sequence correlates well with the residues involved in cluster formation (Fig 7D). In addition, it may be pertinent to note that some of these residues, e.g. A27, L33, V35, Y50, T51, Y56 and V61, belonging to these clusters also showed alternate conformational states at sub-denaturing concentrations of urea (Fig. 4A). We may speculate that these residues of the Dok1 PTB domain have an intrinsic tendency toward alternate conformational states in denaturing conditions. Such intrinsic tendency of alternate conformations of these residues may be linked as a consequence of their high AABUF in the folded state of the PTB domain.

### Implications to the Folding of PTB Domain

The 3D structure of the PTB domain of Dok1 represents the core of the super family of PH and PTB domains. The current study delineates insights towards conformational characteristics and alternate high energy states of an important class of signaling protein. The alternate conformational states of the ligand binding site could be a common occurrence across the IRS-like PTB domain proteins. The residual helical secondary structure of the conserved C-terminal region may be viewed as a potential initiation site for folding of the PTB/PH domains. In addition, the non-random conformations around the adjacent non-polar clusters can initiate hydrophobic collapse during removal of denaturants. Consequently, potential folding initiation site at the early stages of folding may be stabilized from these local hydrophobic collapses and nascent helical secondary structure.

## Materials and Methods

### Protein expression and purification

His tagged human Dok1 PTB domain (Q154 – G256; Swiss-Prot Q99704) was sub cloned into the pET14b vector and over expressed into *Escherichia coli* Rosetta cells, grown at 37°C either in LB or M9 medium containing [^15^N] ammonium chloride and [^13^C] glucose (Cambridge Isotope Laboratories). Cells were induced with 1 mM IPTG at an OD_600_ of 0.6–0.7 and incubated for 6–12 hours at 16°C for protein production. Cells were centrifuged and resuspended in 20 mM Tris-HCl buffer, pH 8.0. Cell solutions were lysed by sonication and the supernatant was applied to Nickel-NTA column for His tag-based affinity purification (Qiagen). The weakly bound proteins were removed from the column by extensive washing with appropriate buffers. Target protein was eluted with buffer containing 500 mM imidazole. Further, purified protein was extensively dialyzed against 50 mM sodium phosphate buffer, containing 100 mM NaCl and 2 mM DTT at pH 6.0. Samples of Dok1 PTB domain were loaded onto a Hiload Superdex 75 16/26 GL preparative column with the AKTA FPLC UPC-900 system (GE Healthcare UK Ltd., England). FPLC column was equilibrated with 50 mM sodium phosphate buffer containing 100 mM sodium chloride and 2 mM DTT, pH 6.0. Protein samples were eluted at a flow rate of 0.5 ml/min and detected spectrophotometrically at 276 nm. The amino acid sequence of Dok1 PTB domain was numbered as Q1- G103.

### NMR spectroscopy

All the NMR experiments were performed on Bruker DRX 600 MHz or 700 MHz spectrometers equipped with cryoprobe. Data acquisition and processing were performed either with Topspin software (BRUKER) or NMRPipe and NMRDraw suite [85], [86] and analyzed by Sparky (T.D. Goddard and D.G. Kneller, University of California, San Francisco). The ^15^N-^1^H HSQC spectra of native and the 7 M urea unfolded state of Dok1 PTB domain were assigned using standard triple resonances NMR methods with a combination of 3-D spectra: HNCA, HN(CO)CA, HNCACB and CBCA(CO)NH [87]. The ^α^H and side chain protons were assigned by 3D ^15^N -NOESY-HSQC (mixing time 120 ms) and 3D ^15^N-TOCSY-HSQC (mixing time 80 ms) experiments in case of urea unfolded sample. NMR spectra were acquired at sample concentrations of 0.6 mM or 1 mM in 50 mM sodium phosphate, 100 mM NaCl, 2 mM DTT and 10% D_2_O at pH 6. For the unfolded state of the protein, all the conditions were same except 7 M urea had been added from a stock solution of 10 M urea made in same buffer. Temperature for all the experiments was set to 298 K. The proton chemical shift was directly referenced to ^1^H resonance frequency of 2,2-dimethyl-2-silapentane-5-sulfonic acid (DSS) whereas the ^13^C and ^15^N dimensions were referenced indirectly. NMR parameters of the unfolded state of Dok1 PTB domain have been deposited to BMRB with accession number 19672.

### Urea titration experiment

Urea titration experiments were recorded at 298 K for Dok1 PTB domain by adding aliquots of denaturant from a 10 M stock made in 50 mM sodium phosphate, 100 mM NaCl and 2 mM DTT at pH 6 to the NMR tube containing ^15^N labelled samples. A series of HSQC spectra were recorded at different urea concentrations ranging from 0.25 M to 7 M. The urea induced chemical shift changes in ^15^N and HN resonances was determined using the following equation which is designated as chemical shift perturbation (CSP), 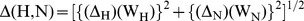
where W_H_ and W_N_ are weighting factors for ^1^H and ^15^N chemical shifts, respectively (W_H_ = 1, W_N_ = 0.154)[56] and Δ_H_ and Δ_N_ are the chemical shift difference for amide proton and ^15^N respectively.

### Temperature dependence of amide proton chemical shift

Temperature dependence of amide proton chemical shift for the Dok1 PTB domain was performed by recording a series of HSQC spectra at seven different temperatures, 285 K, 288 K, 291 K, 294 K, 297 K, 300 K and 303 K. These experiments were done for native and two different concentrations of denaturant, 0.5 M and 1 M urea, in 50 mM sodium phosphate buffer, 100 mM NaCl, 2 mM DTT, pH 6.0. Temperature dependent experiments were also performed at the native condition in the presence of pTyr747 phosphorylated integrin β3 tail at a protein to peptide ratio of 1∶0.6. For each amide proton, a straight line was fitted to the chemical shift temperature dependence and the residuals were plotted to observe the curved temperature profiles.

### Relaxation measurement

The longitudinal (R_1_ = 1/T_1_) and transverse (R_2_ = 1/T_2_) relaxation rate constants of ^15^N labeled Dok1 PTB domain were determined by collecting a series of ^1^H-^15^N HSQC spectra at different time interval with sensitivity enhancement in a Bruker DRX 600 MHz spectrometer at 298 K temperature [88]. Relaxation experiments were recorded for the Dok1 PTB domain at 7 M urea concentration where the protein was in the unfolded condition. The relaxation delays for T_1_ measurement were set to 5, 40, 80, 130, 210, 330, 470, 630, 800, 1000 and 3000 ms with repeating the experiments at the time points of 80 ms and 470 ms for error estimation. For T_2_ measurement the data were acquired with delays of 14.4, 28.8, 43.2, 57.6, 72.0, 86.4, 100.8, 115.2, 129.6, 144.0 and 158.4 ms with the duplicate points at 72.0 ms in this case. T_1_ and T_2_ relaxation experiments were repeated twice for all the experimental conditions and a total of 2048 complex data points with 128 complex increments were collected. Relaxation data were also collected for the natively folded PTB domain in 50 mM sodium phosphate buffer, 100 mM NaCl, 2 mM DTT, pH 6.0. The relaxation rate constants were determined by fitting the cross peak intensities to a mono-exponential function as in I = A*exp(R*t), where I is the signal intensity, A is the signaling constant, R is the relaxation constant and t is the time point [89], [90]. The steady state ^1^H-^15^N hetero-nuclear NOE values were determined by using a pair of spectra recorded with and without proton saturation. The NOE experiment with proton saturation was carried out with a 2 s inter-scan relaxation delay followed by 3 s of proton saturation. Similar delays were used for the heteronuclear NOE experiments of the unfolded proteins in urea[26], [35]. The ^1^H-^15^N hetero-nuclear NOE experiments spectrum were recorded with 2048 complex data points with 256 complex t_1_ points. The NOE values were determined as a ratio of peak intensities with and without proton saturation. A reduced spectral density analysis of the ^15^N relaxation data was carried out for the 7 M urea unfolded state of the Dok1 PTB domain as described earlier[91], [92].

## Supporting Information

Figure S1 **A bar diagram showing combined chemical shift difference of ^15^N and HN resonances of each residue of the PTB domain between native and 1M urea.** A line drawn at 0.03 ppm indicates average changes of the chemical shift.
(TIF)

Figure S2 **Chemical shift changes of selected amide proton resonances with temperature.** Sections of ^15^N-^1^H HSQC crosspeaks of residues showing curved (V61, R69, G85, Q84) and linear (R8, G57, A67, marked as control) temperature dependence. ^15^N-^1^H HSQC spectra were obtained at temperatures of 285 K (red), 288 K (light red), 291 K (green), 294 K (yellow), 297 K (purple), 300 K (cyan) and 303 K (pink) at 0 M, 0.5 M and 1 M urea and in presence of tyrosine phosphorylated β3 tail peptide.
(TIF)

Figure S3 **Urea-protein NOEs.** Slices of 3D ^15^N-edited NOESY-HSQC spectra of the PTB domain showing NOEs between urea protons resonating at 5.9 ppm with the amide protons of representative residues of the PTB domain in 7 M urea.
(TIF)

Figure S4 **Relaxation parameters of the native PTB domain.** Bar diagram showing R_1_ (panel A), R_2_ (panel B) and heteronuclear NOE (panel C) of the native PTB domain as a function of residue.
(TIF)
