# Supplementary figures and images for: NMR Characterization of the Near Native and Unfolded States of the PTB Domain of Dok1: Alternate Conformations and Residual Clusters
Source: PLoS One. 2014 Feb 28;9(2):e90557. doi: 10.1371/journal.pone.0090557 (PMC3938774; doi:10.1371/journal.pone.0090557)

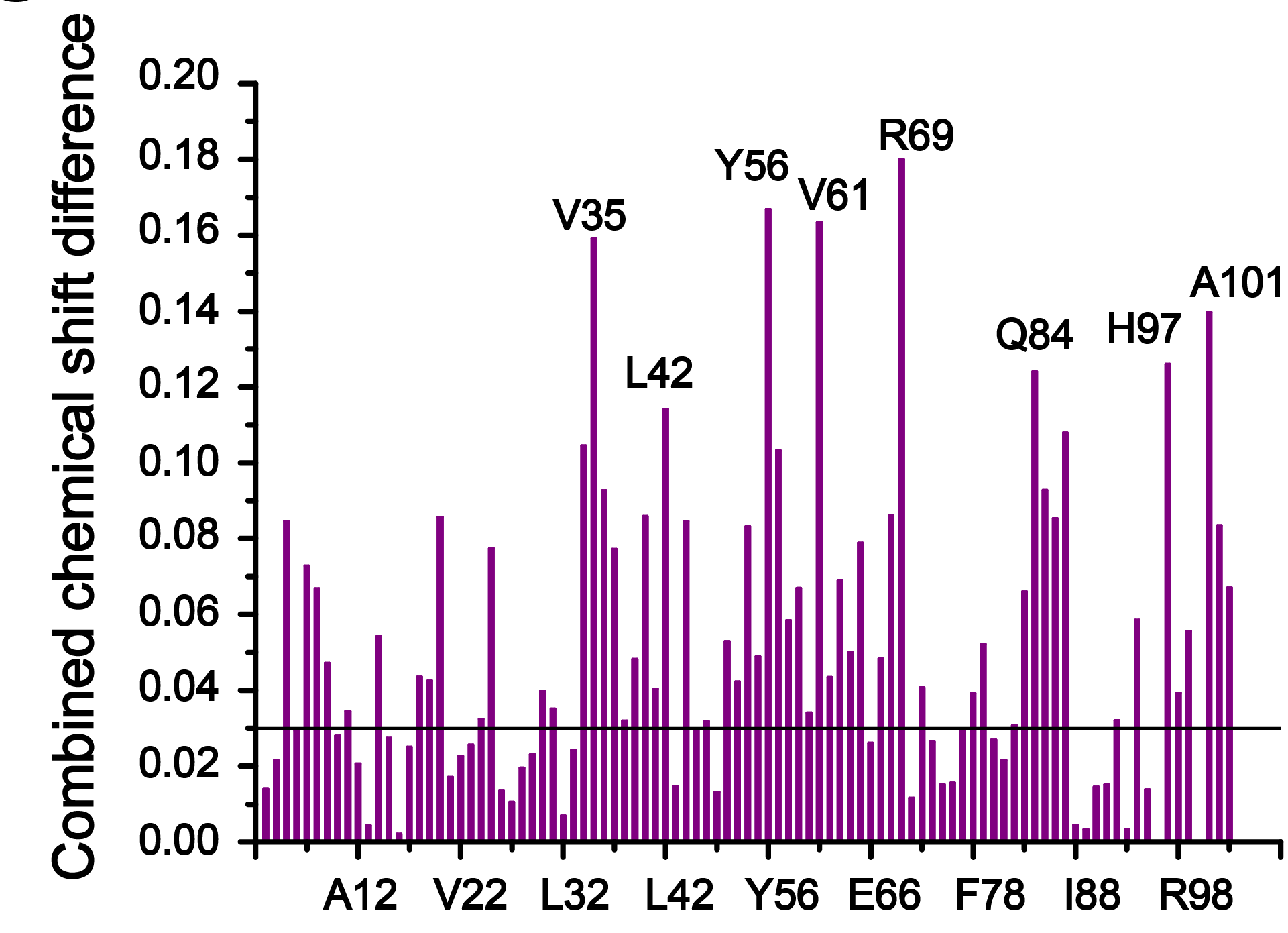

Supplement: Figure S1 — A bar diagram showing combined chemical shift difference of 15N and HN resonances of each residue of the PTB domain between native and 1M urea. A line drawn at 0.03 ppm indicates average changes of the chemical shift. (TIF) [file pone.0090557.s001.tif]

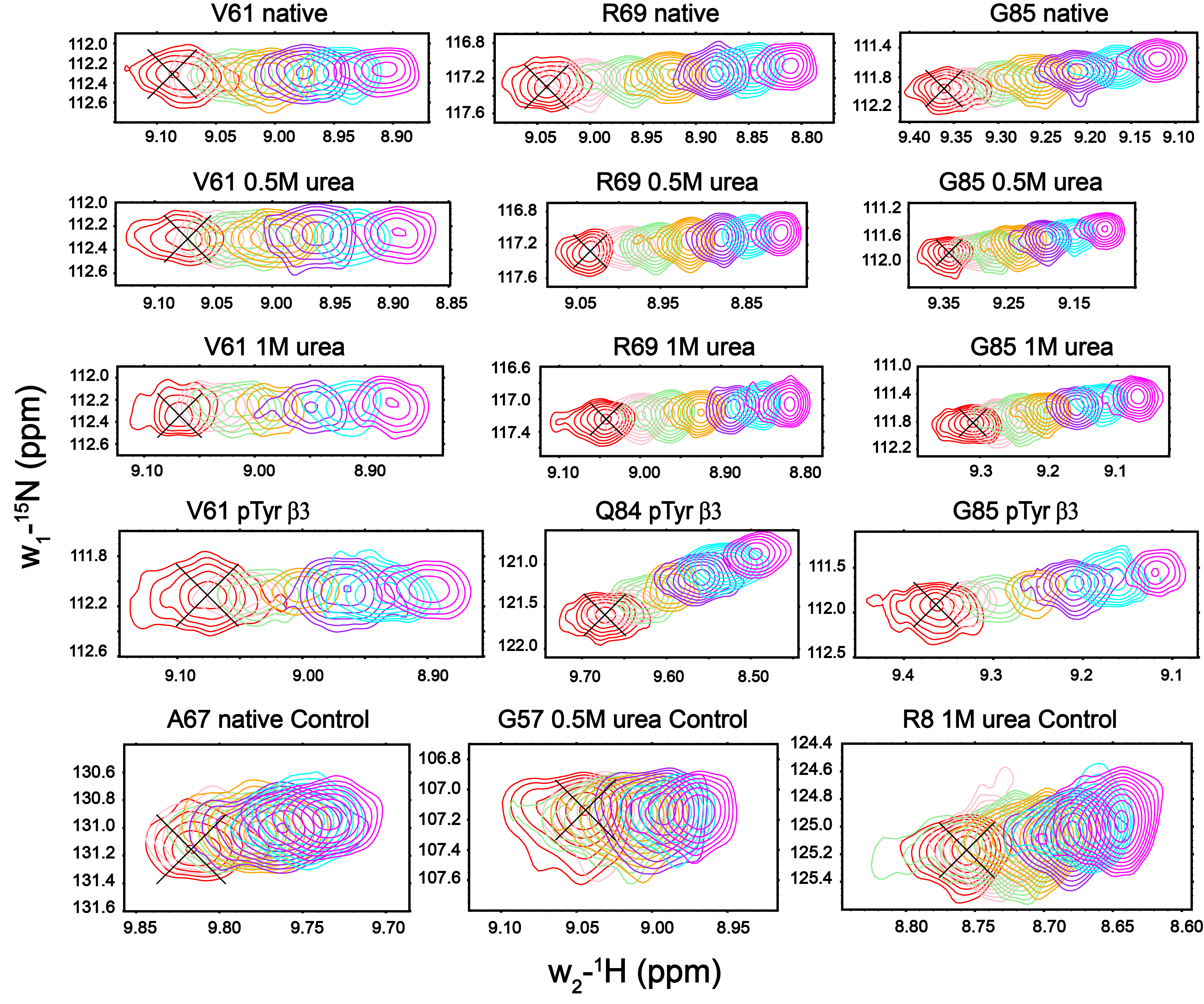

Supplement: Figure S2 — Chemical shift changes of selected amide proton resonances with temperature. Sections of 15N-1H HSQC crosspeaks of residues showing curved (V61, R69, G85, Q84) and linear (R8, G57, A67, marked as control) temperature dependence. 15N-1H HSQC spectra were obtained at temperatures of 285 K (red), 288 K (light red), 291 K (green), 294 K (yellow), 297 K (purple), 300 K (cyan) and 303 K (pink) at 0 M, 0.5 M and 1 M urea and in presence of tyrosine phosphorylated β3 tail peptide. (TIF) [file pone.0090557.s002.tif]

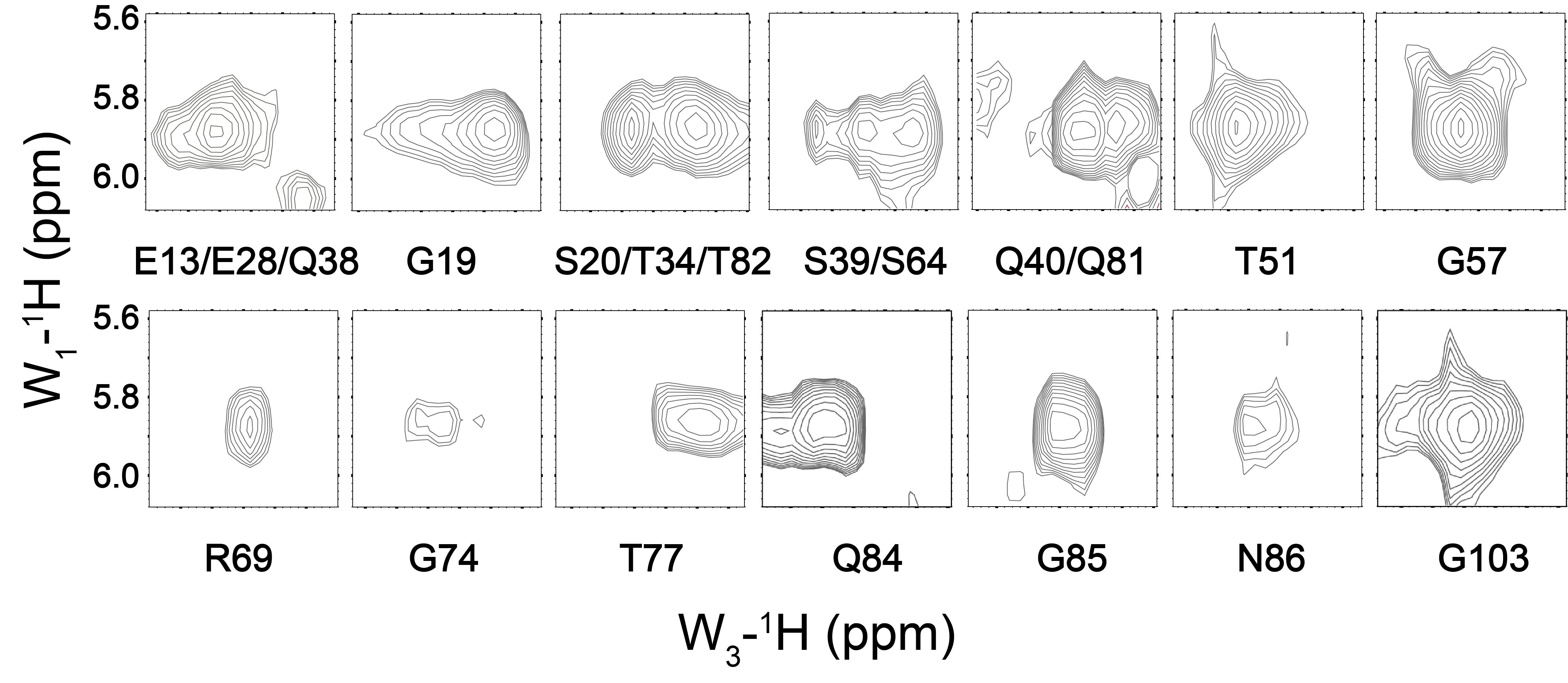

Supplement: Figure S3 — Urea-protein NOEs. Slices of 3D 15N-edited NOESY-HSQC spectra of the PTB domain showing NOEs between urea protons resonating at 5.9 ppm with the amide protons of representative residues of the PTB domain in 7 M urea. (TIF) [file pone.0090557.s003.tif]

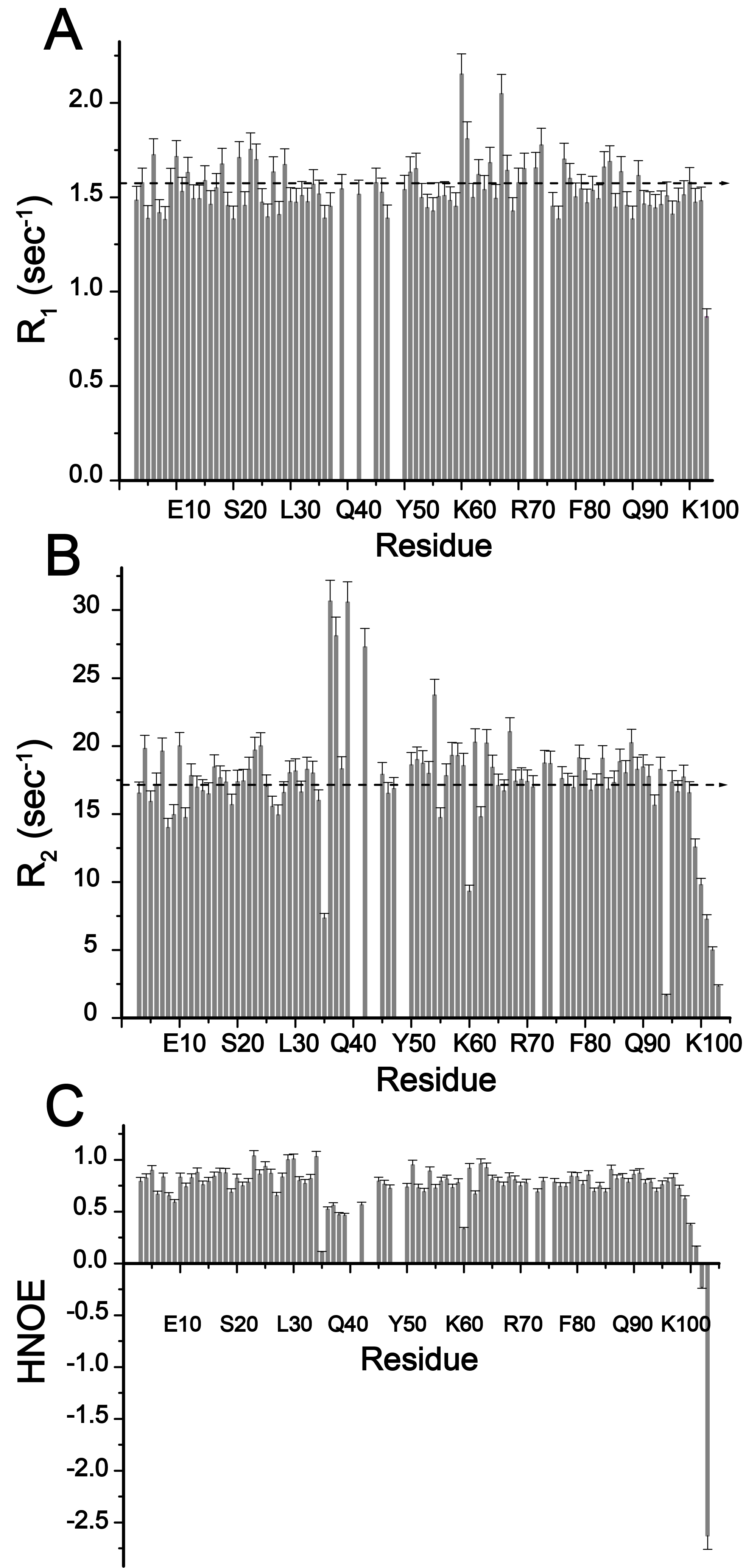

Supplement: Figure S4 — Relaxation parameters of the native PTB domain. Bar diagram showing R1 (panel A), R2 (panel B) and heteronuclear NOE (panel C) of the native PTB domain as a function of residue. (TIF) [file pone.0090557.s004.tif]
